# Fifty Years of Bird Ringing Reveal Opposing Seasonal Responses of Migration Timing to Temperature and Rainfall on Hilbre Island

**DOI:** 10.1002/ece3.73377

**Published:** 2026-04-05

**Authors:** Danielle L. Hinchcliffe

**Affiliations:** ^1^ School of Biological and Environmental Sciences Liverpool John Moores University Liverpool UK

**Keywords:** climate change, conservation, ecology, evolution, ornithology, phenology

## Abstract

Understanding how populations respond to environmental variability is crucial for interpreting long‐term monitoring data, especially for migratory species with complex life cycles that are susceptible to changing weather conditions and may show variation in stopover behaviour. Environmental pressures can influence the timing and duration of migratory stopovers, which may affect local species composition and capture patterns at observatories. This study analyses a 50‐year bird ringing dataset from Hilbre Bird Observatory, a long‐term monitoring site on England's western coast that offers valuable insights into migratory bird patterns. Findings show that Hilbre Island has become warmer and wetter over time, and that the interaction of temperature and rainfall influences observed migration timing. While warmer conditions at the observatory coincide with earlier arrivals, concurrent increases in rainfall can delay arrivals, illustrating how local weather conditions affect patterns of capture. Seasonal ecological differences in residency were observed: in spring, warmer and wetter conditions result in shorter stopovers, whereas in autumn, the same conditions lead to longer stays. Analyses of abundance revealed species‐specific variation, but no significant relationships were detected between local weather variables and overall capture numbers. Overall, the study highlights the complex and sometimes opposing effects of temperature and precipitation on bird migration and residency at Hilbre and emphasises the value of long‐term ringing data for detecting these patterns. The findings underscore the importance of considering weather‐related sampling limitations when interpreting observatory data and provide a foundation for further studies on how local conditions influence observed migratory behaviour.

## Graphical abstract

This study analyses 50 years of bird ringing data from Hilbre Bird Observatory to investigate how local weather affects migratory timing and stopover behaviour. A key novel finding is the interactive effect of temperature and rainfall—warmer conditions tend to advance arrivals, but simultaneous increases in rainfall can delay them, revealing opposing weather influences on migration. These results highlight the complexity of climate impacts on bird movement and underscore the value of long‐term ringing data for uncovering nuanced, weather‐driven behavioural patterns.

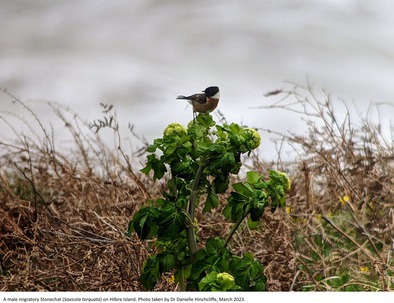

## 1 Introduction

Recent climate change has altered patterns of abiotic factors that are crucial in shaping species abundance and geographic distribution, as well as their variability over time (IPCC 2022; Anderson et al. 2023; Germain et al. 2023; Hickinbotham et al. 2025). If a species is unable to respond in real time to novel environmental conditions, they will endure population declines and be at risk of extinction (Thomas et al. 2004). In migratory species, the timing of key life‐history events—such as arrival at breeding or feeding grounds—is critical for survival and reproductive success in environments that are seasonally variable. Life‐history theory predicts that individuals will evolve traits that best balance fitness costs and benefits, given environmental constraints (Lack 1968). Therefore, adjusting the timing of important life‐history events that is, phenology (such as migration) in response to seasonal and environmental changes, is a thoroughly documented consequence of climate change (e.g., Cleland et al. 2007; Van Buskirk et al. 2009).

Studies have shown that migratory birds can rapidly advance spring migration timings in response to climate change (Lehikoinen et al. 2004; Møller et al. 2008; Romano et al. 2023), and the predominant driver for this adaptation is to avoid “trophic mismatch” that is, ensuring arrival timings coincide with peak food availability (Visser et al. 1998; Both and Visser 2001; Visser et al. 2012). However, this should not be accepted a simple uniform response by migratory birds because it overlooks important complexities. There is considerable variation among species and populations, as different geographic regions or habitats exert different local environmental conditions and selective pressures (e.g., species‐specificity—Both et al. 2006; geographic variation—Møller et al. 2008). Phenological shifts can also be driven by multiple factors beyond climate. Birds rely on many different cues for timing their migration that can all vary in how they shift with climate change, such as day length (e.g., Pine siskins change their spring migration in response to solely increasing day length—Robart et al. 2018), temperature (e.g., Asian houbara time their spring departures based on temperature consistencies—Burnside et al. 2021), and resource availability (e.g., Red‐backed shrike, Common cuckoo and Thrush nightingale all track vegetation greenness across continents and adjusting migration timing/routes to match shifting food peaks—Thorup et al. 2017).

Migration timing is a complex trait influenced by a suite of ecological, environmental and evolutionary factors that vary widely. Studying this variation helps us understand species' vulnerabilities and resilience to ongoing climate change. Monitoring migratory birds from a fixed location in the UK that regularly hosts diverse migratory species offers several scientific and conservation advantages, especially when the data collection is standardised—as is the case with bird observatory bird ringing activities that catch birds via mist‐netting techniques at “constant effort sites” (Gregory et al. 2004). Having consistency over time allows the detection of subtle yet reliable long‐term abundance and population trends and species composition. Furthermore, we can then link such trends to local climate or habitat changes and compare data with other fixed monitoring sites to explore spatial variation in migration responses alongside temporal variation. This helps us to better understand declines in migrant species and supports targeted conservation actions, as well as identifying changes that may simply reflect climate‐influenced sampling bias.

It is important to make use of dedicated bird observatories set up along migration flyways, as they typically obtain phenological data, either through trapping and/or by direct observation (e.g., Hüppop and Hüppop 2003). Hilbre Island, although small (approximately 6 ha), lies on the East Atlantic Flyway, a major migration route for birds travelling between Europe, Africa and the Arctic (Figure S1), and its location at the mouth of the Dee Estuary makes it a natural stopover site for migratory passerines, waders, and seabirds to rest and refuel. Despite its size, long‐term observations and ringing data demonstrate its consistent use by migrants: the Hilbre Bird Observatory, established in the late 1950s (53.383° N, 3.233° W), has recorded over 1000 individual birds annually, with a total of 223 species documented, providing one of the longest unbroken datasets of passerine migration in the region (Wirral Council 2024). Observations indicate that migratory passerines feed on the invertebrate populations of the island plateaux before continuing their journeys, highlighting its functional role as a stopover habitat. The ecological importance of Hilbre Island is recognised through multiple biodiversity designations based on its location on the Dee Estuary, including Site of Special Scientific Interest (SSSI), Special Protection Area (SPA), Special Area of Conservation (SAC), and Ramsar Wetland of International Importance, reflecting its value for both wintering waders and staging migratory birds (Cheshire Now 2005; Wirral Council 2024). Standardised mist‐netting protocols at constant effort sites enable consistent data collection and comparison across years and sites (BTO 2004). Nevertheless, local conditions and practical constraints mean some variation in effort and detectability persists. As a result, ringing data should be treated cautiously, with awareness of these inherent limitations.

Using this dataset we investigate how local climatic variation, specifically temperature and rainfall, affects patterns of bird migration and stopover behaviour at Hilbre Island. We hypothesise that warmer spring temperatures will coincide with earlier arrival dates, but increased rainfall may delay arrivals by creating poor foraging or migratory conditions. Peak migration timing may follow similar patterns, though likely less sensitive to short‐term weather fluctuations. We further predict that birds will adjust their residency at the observatory in response to weather, staying longer during adverse conditions and departing more quickly when conditions are favourable, as these behavioural adjustments are likely mediated by refuelling rates. Poor weather can reduce prey availability or foraging efficiency, slowing energy gain and prolonging stopovers, whereas favourable conditions facilitate rapid refuelling and earlier departure. Finally, we expect that local capture rates, as a proxy for abundance at the observatory, may vary with weather conditions, reflecting both true changes in bird presence and weather‐related capture bias. For instance, rainfall and strong winds can limit mist‐netting opportunities or reduce capture efficiency, leading to lower apparent abundance even when birds are present in similar numbers. Thus, we predict that weather will influence observed capture rates through both biological responses and sampling bias, and that these effects may differ among species depending on their behaviour and habitat use. By explicitly linking these predictions to our modelling approach, we aim to quantify how local weather interacts with species‐specific migration strategies and seasonal dynamics to influence observed phenology and residency on Hilbre Island.

## 2 Materials and Methods

### 2.1 Data Acquisition and Availability

Standardised bird ringing data were obtained from the DeMON Database (BTO 2024), which stores British Trust for Ornithology (BTO) ringing records submitted by local bird ringers with Constant Effort Site (CES). This is part of a national monitoring scheme designed to track temporal and spatial changes in breeding bird populations. The CES operates under strict protocols to ensure consistency across years and locations. Birds are caught in mist nets set up in fixed positions and operated for the same number of visits and duration each year. All birds captured were ringed or identified if already ringed, and biometric data including species, age, sex, wing length and weight were recorded. We used data from 1971 up to 2017, as that is what was provided by Hilbre Bird Observatory and matched with our available weather data, used from the publicly available HadUK‐Grid Climate Dataset, which covers all UK land area at 1 km x 1 km resolution (Met Office et al. 2021). Data on rainfall and temperature were taken for the grids covering Hilbre Island. Extracted data included daily, monthly and yearly values for maximum, minimum and average measures. Annual snowfall data was also extracted. All data and code used in this study are stored in a repository and are available upon request.

### 2.2 Climate Models

To examine how the climate has changed at Hilbre, three models were run, each with “year” as the numeric predictor of (a) average rainfall, (b) average temperature and (c) average snowfall (each model spanning 25 years of climatic data). Both the rainfall and temperature models were fitted with a Gaussian error structure; skew in the snowfall data meant that this model was fitted with a log‐normal error distribution.

### 2.3 Phenology Models

To examine climatic impacts on migratory patterns, the impact of a changing environment was modelled on the earliest day within spring (period defined as Julian day 60–152) and autumn (Julian day 212–304) that each species was observed within a given year. The relationship between climate and the Julian day of peak capture for each species was also modelled.

For the phenology analyses, only species with at least fifty capture records across the entire dataset were included from the total species list (Table S1). Although hundreds of species were initially available, many had records concentrated in only a short portion of the timeline, limiting their utility for long‐term trend analysis. Arrival and peak capture timing differ widely among species, reflecting different migratory phenologies and seasonal windows. To make timing comparable across species and to account for these different seasonal ranges, we normalised the earliest arrival day and day of maximum capture within each species to a 0–1 scale, where 0 represents the earliest recorded day and 1 represents the latest recorded day for that species. Normalising in this way also allows the response to be modelled using a zero–one inflated beta distribution, which is appropriate for bounded proportional data and accommodates observations at the extremes of the seasonal range.

In all spring and autumn models, annual sampling effort for each species was included as a control variable. Sampling effort was expressed as the proportion of total capture events in a year that involved a given species. This was included because even in nominally “constant effort” ringing schemes, effective sampling effort varies subtly among years and species due to weather/access variability, operational inconsistencies, capture bias and temporal coverage. Including a sampling‐effort covariate acknowledges residual variation in effective effort that can influence capture probability and, by extension, timing metrics like first or peak capture day. The predictors were climatic variables, that is, average UK‐wide rainfall and temperature across the 90 days preceding the earliest day of arrival within each season. Snowfall was not included, as only yearly rather than monthly values were available for this variable. While we recognise that migration duration varies among species, a 90‐day window was selected as a conservative representation of the period encompassing both pre‐departure conditioning and migration for the majority of species considered. Climatic conditions over this broader temporal scale are likely to influence migratory decisions indirectly through cumulative effects on resource availability, physiological condition and habitat suitability, rather than through short‐term weather events alone (e.g., Jenni and Kéry 2003; Fiedler et al. 2004; Haest et al. 2020). The use of a multi‐month climatic summary is therefore intended to capture these integrated seasonal influences, while acknowledging that alternative, shorter windows (e.g., 30 or 60 days) may reflect different aspects of climatic drivers and represent a potential avenue for future investigation.

UK‐wide climatic predictors were used because, although many species migrate from regions beyond the UK, national‐mean temperature and rainfall provide integrative indicators of broad‐scale climatic conditions correlated with continental weather systems influencing migration into the UK. This approach offers a consistent proxy for large‐scale environmental trends affecting arrival timing, while avoiding the data gaps and heterogeneity of multi‐regional datasets. Local measures were not used, as migratory timing is driven mainly by broad‐scale climatic cues rather than site‐specific conditions. Moreover, climatic data from Hilbre Island were highly correlated with UK‐wide means (Figure S4), confirming that national averages robustly capture local temporal trends relevant to migration. Since both temperature and rainfall may have synergistic effects on bird migratory behaviour, an interaction was included between these variables in the model. In all models, species were included as random effects (with random slopes for all predictor variables) to account for multiple observations of the same species and potential within species responses to climate; in addition to including a random intercept for year (as a categorical variable) to account for any within sampling year impacts not included among fixed effects. Models of earliest arrival times in spring/autumn were fitted using a zero‐inflated beta error distribution; models for maximum number of arrivals in spring/autumn were fitted using a Gaussian error distribution.

### 2.4 Residence Length Analyses

To further explore seasonal dynamics, post hoc analyses were carried out to examine the length of seasonal residence in spring and autumn. For each species in each year, residence length was calculated as the difference between the latest and earliest Julian day of observation within that season. Years in which a species was observed only once (yielding a residence length of zero) were excluded from analysis. Because species differed in their absolute ranges of seasonal residence duration, residence lengths were normalised within each species and season to facilitate cross‐species comparison. After filtering, the final dataset comprised 609 species–year observations in spring and 432 species–year observations in autumn. The fixed and random effect variables in these models were the same as in our spring and autumn phenology models. Since we were using a normalised response variable, models were fitted with a zero–one‐inflated beta error distribution.

### 2.5 Bayesian Model Fitting and Validation

For the analyses, a Bayesian approach was employed with all models fitted and estimated using Hamiltonian Monte Carlo methods and Stan software (Stan Development Team 2021) with the *brms* package (Bürkner 2018) within R (R Core Team 2022).

Prior to model fitting, any potential issues of covariation or collinearity between our fixed effects were inspected via pairwise plots, pairwise correlations and variance inflation factors (VIFs). Pairwise plots and correlation coefficients were generated using the ‘covees’ function of the *GGally* package (Schloerke et al. 2024); VIFs were generated using the ‘VIF’ function of the *car* package in R (Fox and Weisberg 2019). For the phenology models, rainfall and temperature were collinear with each other but were fitted in an interaction as could act synergistically on bird migratory behaviour. Subsequent model validations (see below) suggested no issues with this approach.

All continuous predictors were standardised (*z*‐scored: mean = 0, SD = 1) prior to analysis. Accordingly, we used weakly regularising priors for the fixed effects (β ~ Normal(0, 1)), which are appropriate for standardised predictors and improve model stability without constraining plausible effect sizes (Gelman et al. 2008). For the priors for the components of the random effects, we used the default priors provided by the ’get_prior’ function of *brms*, namely a weakly regularising half student‐t prior (df = 3, scale parameter = 10) for the random intercepts, and a uniform LKJ Cholesky prior (*η* = 1) for covariance matrices of the random slopes. The choice of model family and error structure was guided by the distributional properties of the response variables. For each response variable, we inspected residual distributions and posterior predictive checks to confirm that the selected family provided an adequate fit. Alternative error structures were evaluated where relevant and the model family that provided the best fit without evidence of overdispersion was retained. For all models, we specified three chains of 5000 iterations, 2000 of which were devoted to the warm‐up. Sampling diagnostics (Rhat < 1.01) and trace plots confirmed chain convergence for all models. Effective sample sizes confirmed no issues with autocorrelation of sampling for all models.

To interpret the strength and uncertainty of the associations between predictor variables and outcomes, the model estimate is reported along with 90% credible intervals and the proportion of posterior (*p*+ or *p*−) supporting the direction (positive or negative) of the model estimate of the associations (Martin et al. 2020; McElreath 2020; McShane et al. 2019). Effects were well supported if the effect direction was supported by more than 95% of the posterior distribution, and those whose direction was supported by more than 90% of the posterior distribution were weakly supported. All models were validated using posterior predictive checks (see Figures S2 and S3 for plots of these checks).

### 2.6 Abundance Models

For the abundance model, we used annual species‐specific capture rates, defined as the number of captures of each species in a year divided by the total number of captures of all species in that year. This metric represents the relative proportion of each species in the total capture sample, and thus accounts for variation in overall sampling effort and session frequency across years. Only species with ≥ 50 captures in a given year were included, and only species that could be classified as resident and/or migratory at Hilbre during the relevant season were retained for analysis.

Capture rates varied substantially between species, so this variable was normalised within species between 0 and 1. For the analysis, three‐way interactions were fitted between our climatic variables (yearly rainfall and temperature) and the migratory status of the species (three levels: resident, migratory, mixed status). However, no meaningful association was found between these interactions and annual abundance rates, and therefore, ran the model with only the single terms included (climatic variables and migratory status of the species; rainfall and temperature were only weakly correlated indicating both could be included as fixed effects). Again, species identity was included as a random intercept, with random slopes for our climatic variables to account for multiple observations of the same species and potential within‐species responses to climate.

In addition, to examine long‐term trends in relative abundance, a model was fitted with year (numeric) as a predictor of normalised abundance. Given the response variable was continuous and bounded between 0–1, and included exact 0 and 1 values, models were fitted with a zero–one‐inflated beta error distribution.

## 3 Results

Between 1971 and 2017, a total of 41,231 capture records representing 29,338 individual birds and 41 species were recorded. Annual capture totals averaged 877 records per year (range 156–2251), reflecting variation in sampling effort among years. The most frequently captured species were WILWA (10,521 records), MEAPI (5581), ROBIN (3273), GOLDC (3256) and LINNE (3098). All 41 species were retained for abundance analyses, while 32 and 34 species met inclusion criteria for spring and autumn phenology analyses, respectively.

From 1971 to 2017 on Hilbre Island, rainfall has increased over time (*p*+ =0.921), with an estimated increase of 0.13 mm per year, along with temperature (*p*+ =1.0), which has had an estimated increase of 0.03°C per year in the same period. We do not find any strong evidence that snowfall is changing over time, but trends suggest a slight decline (p‐ = 0.735; Table S2; Figure S4).

### 3.1 Phenology

**FIGURE 1:**
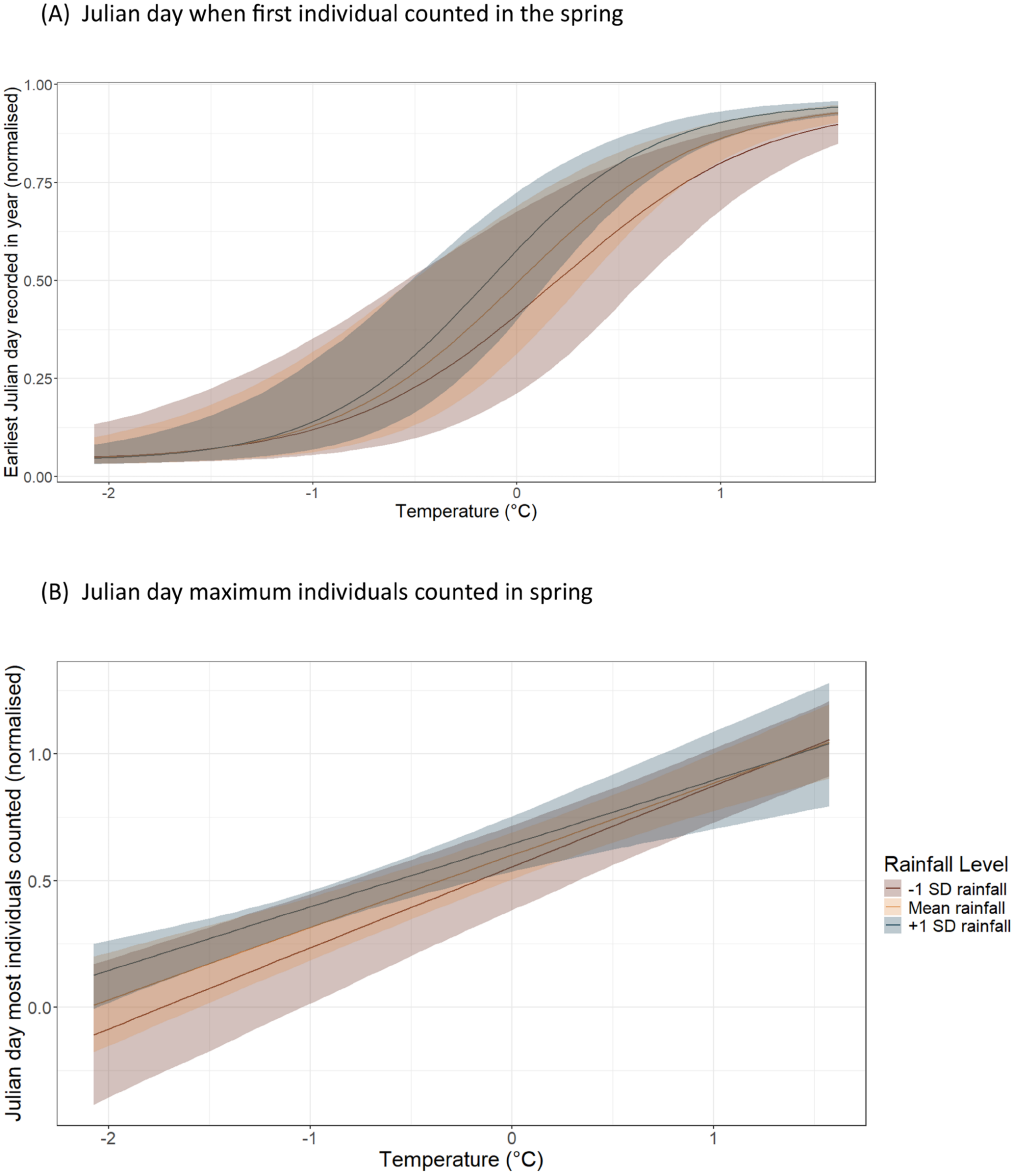
The relationship between average temperature (°C) and rainfall (mm) with (A) earliest arrival day within spring and (B) day when maximum number of individuals were counted in spring in each species. Y‐axis is normalised from 0 to 1, corresponding to Julian days 60–151 (March 1st to May 31st). Lines show the population‐level model predictions (mean random effects) of temperature under different rainfall scenarios: −1 SD, mean, +1 SD rainfall. Rainfall was fitted as a continuous variable and is only categorised for visual purposes. Shaded ribbons show 90% credible intervals. (A) Julian day when first individual counted in the spring. (B) Julian day maximum individuals counted in spring.

Temperature appears to be the strongest and most significant climate predictor of earlier spring migration. There was a clear and consistent association between the interaction between average temperature and rainfall and the earliest Julian day of arrival in spring (*p*+ =1.000; Table 1; Figure 1), wherein warmer and wetter springs were associated with later arrival dates. Species‐level random effects revealed substantial heterogeneity in earliest arrival timing, with considerable variation in baseline arrival dates among species (random intercept SD = 2.43) and in species‐specific responses to temperature (SD = 1.47) and rainfall (SD = 1.00). This interaction between temperature and rainfall was less pronounced for the Julian day corresponding to the maximum number of individuals captured across species (*p*− = 0.981; Table 1; Figure 1). Warmer springs were generally associated with increased arrivals. However, when considering peak migration timing, this effect was primarily evident in wetter springs. In contrast to earliest arrivals, species‐level variation in peak timing was comparatively small (random intercept SD = 0.22; temperature slope SD = 0.13; rainfall slope SD = 0.23), indicating that peak spring migration timing was more consistent across species. Year‐level variation was minimal in both models (SD = 0.048 and 0.009 for earliest and peak models, respectively).

**TABLE 1:** Model results exploring how climatic factors related to (a) earliest arrival day in spring, (b) day of maximum number of arrivals in spring, (c) earliest day of arrival in autumn and (d) day of maximum arrival in autumn for migratory birds on Hilbre.

Coefficient	Estimate	Error	Lower 95% CI	Upper 95% CI
***(a) Spring: Earliest arrival***				
Intercept	−0.0.01	0.45	−0.85	0.88
**Temperature**	**2.19**	**0.28**	**1.62**	**2.72**
Rainfall	0.35	0.20	−0.05	0.76
Sampling effort	0.13	0.37	−0.70	0.86
**Temperature**: **Rainfall**	**0.786**	**0.003**	**0.18**	**0.31**
***(b) Spring: Max # arrivals***				
Intercept	0.53	0.05	0.44	0.62
**Temperature**	**0.28**	**0.04**	**0.20**	**0.36**
Rainfall	0.05	0.06	−0.07	0.15
**Sampling effort**	**1.90**	**0.60**	**0.72**	**3.10**
**Temperature: Rainfall**	**−0.03**	**0.02**	**−0.07**	**−0.00**
***(c) Autumn: Earliest arrival***				
Intercept	0.53	0.05	0.44	0.62
**Temperature**	**0.28**	**0.04**	**0.20**	**0.36**
Rainfall	0.05	0.06	−0.07	0.15
**Sampling effort**	**1.90**	**0.60**	**0.72**	**3.10**
**Temperature: Rainfall**	**−0.03**	**0.02**	**−0.07**	**−0.00**
***(d) Autumn: Max # arrivals***				
Intercept	0.53	0.04	0.44	0.62
**Temperature**	**0.28**	**0.04**	**0.20**	**0.35**
Rainfall	0.04	0.05	−0.06	0.15
**Sampling effort**	**1.88**	**0.62**	**0.68**	**3.13**
**Temperature: Rainfall**	**−0.03**	**0.02**	**−0.07**	**−0.00**

*Note:* Coefficients in bold had estimate directions supported by > 95% of the posterior distribution.

**FIGURE 2:**
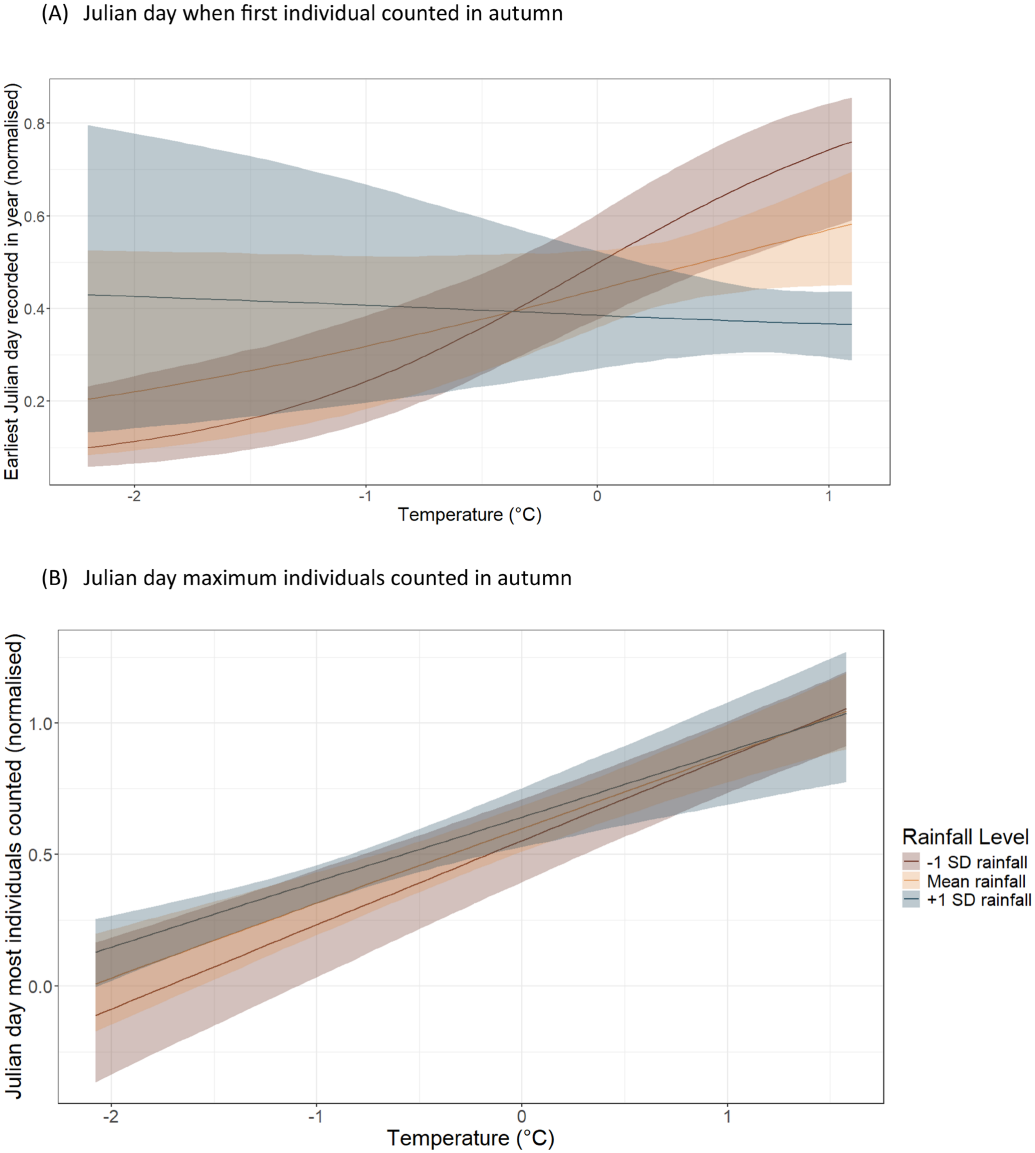
The relationship between average temperature (°C) and rainfall (mm) with (A) earliest arrival day within autumn and (B) day when maximum number of individuals were counted in autumn in each species. Y‐axis is normalised from 0 to 1, corresponding to Julian days 213–305 (August 1st to October 31st). Lines show model‐predicted effects of temperature under different rainfall scenarios: −1 SD, mean, +1 SD rainfall. Rainfall was fitted as a continuous variable and only categorised for visual purposes. Shaded ribbons show 90% credible intervals. (A) Julian day when first individual counted in autumn. (B) Julian day maximum individuals counted in autumn.

Similarly to spring, autumn phenology (both timing and abundance) was strongly shaped by temperature at the population level, with rainfall playing a secondary, moderating role through its interaction with temperature (Table 1; Figure 2). This pattern was evident for both the earliest Julian day of arrival in autumn (*p*− = 1.000) and the Julian day corresponding to the maximum number of individuals captured across species (*p*− = 0.980). For earliest arrivals, species‐level random effects indicated moderate variation in baseline timing among species (random intercept SD = 0.92), along with substantial heterogeneity in species‐specific responses to temperature (SD = 1.72) and rainfall (SD = 1.11). Both measures indicated that warmer and drier years tended to lead to earlier arrival dates and higher peak numbers. In contrast, species‐level variation in peak autumn timing was comparatively small (random intercept SD = 0.21; temperature slope SD = 0.13; rainfall slope SD = 0.23), suggesting greater consistency across species in peak migration timing. Year‐level variation was modest for earliest arrivals (SD = 0.14) and minimal for peak timing (SD = 0.009).

**FIGURE 3:**
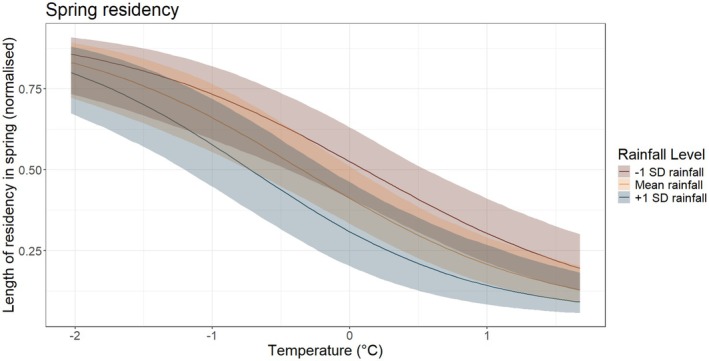
The relationship between the interaction between average temperature (°C) and rainfall (mm) and the length of residency in spring. Y‐axis is normalised from 0 to 1, corresponding to Julian days 60–151 (March 1st to May 31st). Rainfall was fitted as a continuous variable and is only categorised for visual purposes. Shaded ribbons show 90% credible intervals.

For the residency length analyses, warmer and wetter conditions reduced residency in the spring (p = 0.841; Table 2; Figure 3), whereas conversely, they increased residency for autumn migrant birds, favouring those conditions (*p*+ = 0.998; Table 2; Figure 3). Interestingly, the interaction between temperature and rainfall appears to flip direction between seasons: in warmer and wetter years, birds arrive later in spring but stay for less time, whereas in autumn they arrive later but stay for longer. There was substantial variation among species in both baseline residence length and responses to climate. In spring, species‐level standard deviations were 0.79 for the intercept, 0.47 for rainfall, 0.30 for temperature, and 1.17 for effort, indicating considerable differences among species in residency duration and climatic sensitivity. In autumn, intercept variation was smaller (SD = 0.20), but species differed more in rainfall responses (SD = 0.66), with temperature responses showing moderate variation (SD = 0.25). Year‐level variation was smaller in both seasons (spring SD = 0.26; autumn SD = 0.33), suggesting interannual variability is present but generally weaker than species‐level differences.

**TABLE 2:** Model results exploring climatic factors related to residence length in (a) spring and (b) autumn for migratory birds on Hilbre.

Coefficient	Estimate	Error	Lower 95% CI	Upper 95% CI
***(a) Spring***				
Intercept	0.028	0.317	−0.609	0.659
Temperature	−1.158	0.600	−2.347	0.017
Rainfall	0.080	0.556	−1.009	1.142
Sampling effort	0.026	0.983	−1.942	1.928
**Temperature: Rainfall**	**−0.377**	**0.132**	**−0.632**	**−0.121**
***(b) Autumn***				
Intercept	−0.56	0.12	−0.79	−0.33
Temperature	−0.08	0.17	−0.42	0.25
Rainfall	−0.01	0.20	−0.38	0.38
Sampling effort	1.24	0.74	−0.29	2.65
**Temperature: Rainfall**	**0.27**	**0.09**	**0.09**	**0.46**

*Note:* Coefficients in bold had estimate directions supported by > 95% of the posterior distribution.

### 3.2 Abundance

Visual inspection of capture rates (normalised within species) suggested divergent patterns among species in their abundances in recent years (Figures 4A and 4B). However, when the model is fit to include climatic predictors, there were no significant associations observed between abundances and all climatic predictors included in the model (Table 3a). Overall, it seemed climate was not a strong predictor of abundance in this analysis. When looking at general changes over time, there was a clear but extremely weak positive association between year of observation and normalised abundances (Table 3b), with status (migratory or resident) playing no significant role.

**FIGURE 4A:**
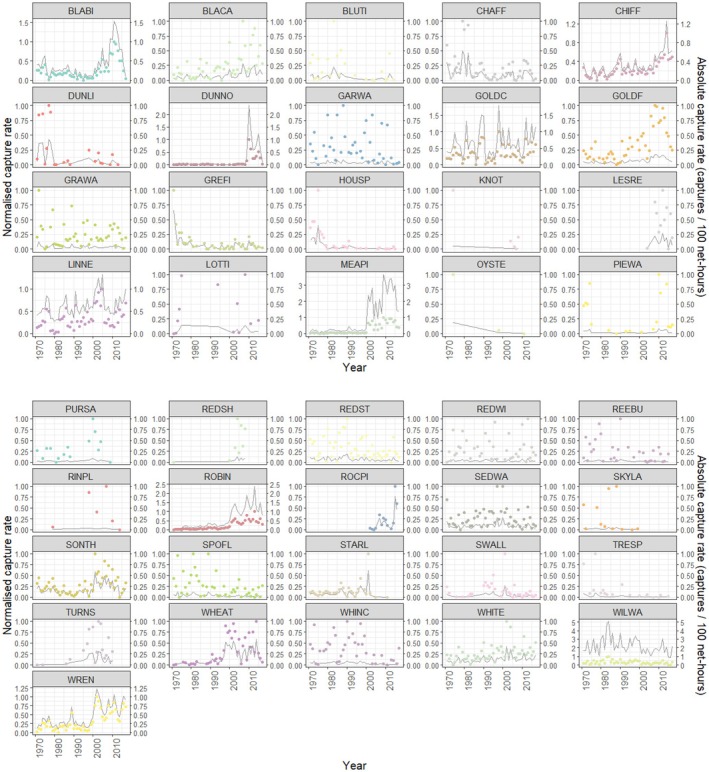
Changes in capture rate on Hilbre Island, 1971–2017, for 41 bird species. Each panel shows annual capture rates for a single species. Coloured points indicate normalised capture rates (unitless) within species to account for large differences in mean abundance. The left y‐axis shows these normalised values, while the darker grey line represents absolute capture rates (captures per 100 net‐hours), obtained by reversing the min–max normalisation. This dual‐axis presentation allows comparison of temporal trends across species while preserving interpretability in real units. Species abbreviations: BLABI (Blackbird), BLACA (Eurasian blackcap), BLUTI (Blue tit), CHAFF (Chaffinch), CHIFF (Chiffchaff), DUNLI (Dunlin), DUNNO (Dunnock), GARWA (Garden warbler), GOLDC (Goldcrest), GOLDF (Goldfinch), GRAW (Grasshopper warbler), GRETI (Great tit), HOUSP (House sparrow), KNOT (Red knot), LESRE (Lesser redpoll), LINNE (Linnet), LOTTI (Long‐tailed tit), MEAPI (Common redpoll), OYSTE (Oystercatcher), PIEWA (Pied wagtail), PURSA (Purple sandpiper), RESDH (Common redshank), REDST (Common redstart), REDWI (Eurasian redwing), REEBU (Reed bunting), RINPL (Ringed plover), ROBIN (Eurasian robin), ROCKPI (Rock pipit), SEDWA (Sedge warbler), SKYLA (Skylark), SONTH (Song thrush), SPOFL (Spotted flycatcher), STARL (Eurasian starling), SWALL (Swallow), TRESP (Tree sparrow), TURNS (Turnstone), WHEAT (Common wheatear), WHINC (Whinchat), WHITE (Common whitethroat), WILWA (Willow warbler), WREN (Eurasian wren).

**FIGURE 4B:**
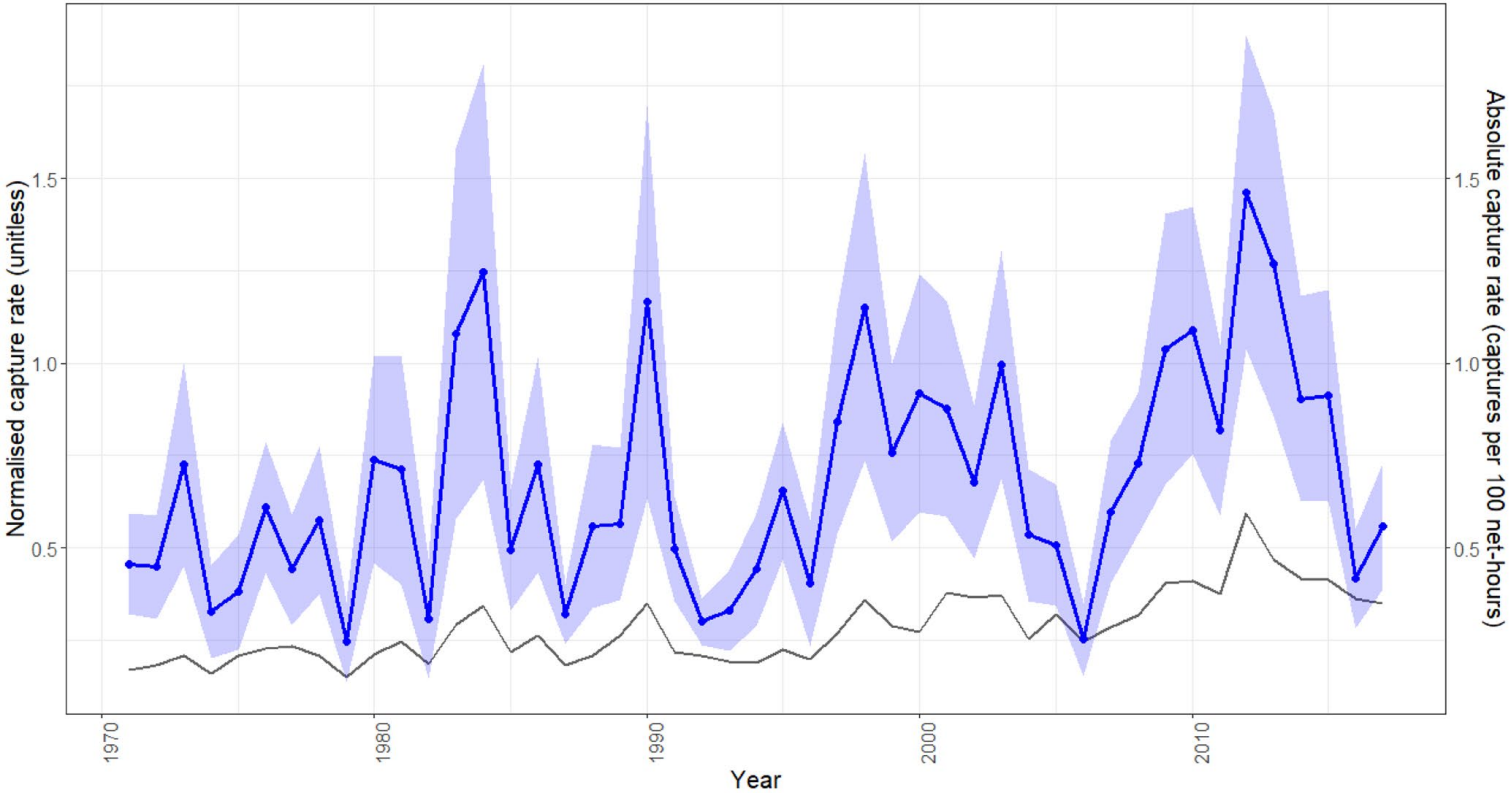
Generalised phenological trend in capture rates of 41 bird species on Hilbre Island between 1971 and 2017. Data are summarised across species, with the mean normalised capture rate shown as a blue line and a 95% confidence interval represented by the shaded blue ribbon. The right y‐axis indicates approximate absolute capture rates derived by reversing the min–max normalisation to retain interpretability in ecological units. The darker grey line shows mean absolute capture rate across species.

**TABLE 3:** Results from model exploring relationships between climatic factors and bird species abundances on Hilbre.

Coefficient	Estimate	Error	Lower 95% CI	Upper 95% CI
(a) Climate as predictor				
Intercept	−1.02	0.08	−1.17	−0.87
Rainfall	0.00	0.03	−0.06	0.07
Temperature	−0.02	0.03	−0.08	0.05
Status (Migratory)				
Mixed	−0.23	0.17	−0.55	0.09
(b) Year as predictor				
Intercept	−18.57	4.23	−26.95	−10.54
Status (Migratory)				
Mixed	−0.24	0.17	−0.57	0.10
**Year**	**0.01**	**0.00**	**0.00**	**0.01**

*Note:* Coefficients in bold had estimate directions supported by more than 95% of the posterior distribution. For the categorical variable, the reference category is in parentheses.

## 4 Discussion

Global climate change and biodiversity loss are profoundly altering ecosystems worldwide, with particularly severe consequences for migratory species and birds, whose survival depends on the stability of habitats and the timing of seasonal cues. Hilbre Island is a relatively small but ecologically rich site, supporting a diverse community of resident and migratory birds and providing important stopover habitat during migration. This biodiversity makes the site particularly useful for detecting ecological responses to environmental change and our results show that, in line with national and global trends, both temperature and rainfall have increased over time. These changes are reflected locally at Hilbre and are associated with shifts in the timing and duration of bird passage, indicating that long‐term ringing data can potentially detect climate‐related changes in local migratory behaviour rather than population‐level phenology.

Temperature was the strongest and most consistent predictor of earlier spring migration, which is well supported in the literature because warmer climates often result in earlier emergence of insects, and so migratory birds need to adjust arrival times to match with peak prey availability (Visser et al. 2010; Saino et al. 2011). Failure to do so results in “trophic mismatch” and has detrimental effects on reproductive success in the subsequent breeding season (Visser et al. 2012; Samplonius et al. 2016; Mallord et al. 2017). However, our findings do not directly assess mismatch on the breeding grounds, but rather the expression of migratory timing at a coastal passage site where arrival patterns are mediated by local and regional weather. At Hilbre Island, it appears that the combination of warmer and wetter springs results in later spring arrival dates. This suggests that weather conditions affecting flight and capture bias at stopover sites may partially obscure the broader phenological signals commonly reported in breeding studies. Excess rainfall may override or complicate the trend of warmer temperatures resulting in earlier arrivals, by creating poor migratory or foraging conditions, delaying resource peaks, or disrupting cues birds rely on (e.g., Schaub et al. 2004; Studds and Marra 2011; Dossman et al. 2023). These patterns highlight the interacting roles of temperature and precipitation in shaping migration timing at regional scales, and the need to consider weather‐mediated capture biases when interpreting long‐term ringing data.

In the UK, wetter springs are often associated with delayed insect emergence and reduced availability of key food resources for migratory birds (e.g., Harrington and Woiwod 2001; Both et al. 2006). High rainfall may also coincide with unfavourable wind conditions, slowing migration and reducing foraging efficiency on route. Together, these effects may counteract the tendency for warmer temperatures to trigger earlier departures from wintering sites, explaining why warmer, wetter conditions can paradoxically result in later arrival dates at Hilbre. In autumn, similar temperature effects were observed, but rainfall exerted a secondary, moderating role, again reflecting the strong influence of weather on local detectability and stopover duration rather than direct migratory scheduling. This seasonal contrast is consistent with general migration theory (Alerstam and Lindström 1990). During spring, birds face strong selection pressure to reach breeding grounds quickly and may be more sensitive to weather that affects migration speed, whereas in autumn, time constraints are relaxed, and birds can afford to migrate more slowly, extending stopovers or waiting for favourable conditions.

Interestingly, the interaction between temperature and rainfall continues to reveal the complexities when trying to understand behavioural responses to environmental variation when considering residency. In spring, warmer and wetter conditions lead to birds having shorter residencies on Hilbre, whereas in autumn the same conditions lead to longer residencies. The interaction effect between temperature and rainfall reverses between seasons, showing seasonal ecological differences.

These contrasting effects likely reflect seasonal differences in migratory strategy and energetic constraints (Alerstam and Lindström 1990; Newton 2008). During spring migration, individuals are generally under stronger time pressure to reach breeding grounds, and warmer conditions may reduce thermoregulatory costs and enhance food availability, facilitating rapid energy accumulation and shorter stopovers despite increased rainfall (Alerstam and Lindström 1990; Hedenström 2008). In contrast, during autumn migration, selection pressures are more closely aligned with energy accumulation and risk avoidance, and warmer, wetter conditions may prolong stopovers by increasing food availability or by constraining foraging efficiency and departure decisions through precipitation‐related effects (Hedenström 2008; Schmaljohann and Eikenaar 2017). From a migration energy perspective, these patterns are consistent with seasonal shifts between time‐minimising strategies in spring and energy‐ or risk‐minimising strategies in autumn.

While the study explored abundance trends, no significant associations with climate predictors were found, suggesting that temperature and rainfall primarily influence timing and residency rather than total capture numbers. However, this absence of correlation should be interpreted cautiously because local capture rates are strongly influenced by weather‐dependent sampling biases. For example, rainfall or strong winds can reduce mist‐netting efficiency or prevent ringing sessions entirely, while species‐specific foraging or movement behaviours may further modulate capture probability. Consequently, some apparent changes—or lack thereof—in abundance may reflect variation in sampling success rather than true shifts in migration phenology or local population size. This highlights the importance of considering these biases when interpreting long‐term trends from observatory data. Overall, while this study's findings partly support the current stance in ornithological literature that a warming climate is resulting in migratory birds arriving at sites earlier, it also presents important nuances showing that local weather conditions can reverse this pattern. Our results therefore capture the complexity of ecological systems, in which animal responses to climate change vary among seasons, sites, and species. Similar results, which oppose typical published trends, are found in other long‐term ringing datasets from UK observatories (Hinchcliffe and Tkaczynski 2025). Publishing such findings is valuable, as they demonstrate that apparent inconsistencies may reflect spatial and methodological differences rather than true biological contradictions.

It is also important to recognise the limitations inherent to observatory ringing data. Phenological interpretations are constrained by weather‐dependent sampling, effort variation, and the selective nature of individuals captured (often juveniles or inexperienced migrants). Capture rates and timing at Hilbre reflect when birds are forced down or choose to stop over under prevailing conditions, not the migratory timing of the wider population. Our hierarchical models treat all species as a single community in order to characterise broad community‐level responses to climate, rather than taxon‐specific patterns. However, subgroup analyses would represent a valuable avenue for future work (Pacifici et al. 2014). Ringing data from coastal observatories are best viewed as indicators of how local weather interacts with migration rather than as direct measures of large‐scale phenological shifts. From a conservation perspective, these findings still hold value. Long‐term observatory datasets provide early warning signals of climate‐linked changes in stopover ecology and can inform the design of adaptive monitoring networks. Future work should integrate ringing data with tracking and citizen‐science records to separate true phenological shifts from local detection effects. Conservation planning should therefore prioritise maintaining such long‐term datasets. This ensures consistent recording of weather and effort variables and the linking of coastal observatory data to inland and breeding‐ground studies to capture the full migratory system.

In summary, Hilbre Island's long‐term ringing record highlights the sensitivity of migratory behaviour to changing temperature and rainfall regimes but also underscores the limits of using passage data to infer population‐level phenological mismatch. By explicitly accounting for weather effects and sampling biases, observatory data can nonetheless serve as an important tool for monitoring how migration timing and stopover dynamics respond to a changing climate and for guiding future multi‐scale studies across the migratory network.

## Author Contributions

**Danielle L. Hinchcliffe:** conceptualization (lead), formal analysis (lead), investigation (lead), methodology (lead), writing – original draft (lead), writing – review and editing (lead).

## Disclosure

Statement of Inclusion: This study uses a long‐term citizen science dataset from the British Trust for Ornithology (BTO) ringing scheme, recognising the invaluable contributions of volunteers in ecological research. We acknowledge the importance of inclusive participation in citizen science and the broader scientific community, valuing contributions from individuals of all backgrounds.

## Conflicts of Interest

The author declares no conflicts of interest.

## Supporting information


**Table S1:** Individual species included in data analysis based on selection criterion described in the methods. Status is based on historical records of bird activity on Hilbre Island. Species in shaded cells appear in all models; the remaining species were included in our analyses of abundance but arrival dates or residence in spring and autumn.
**Table S2:** Model results exploring climate change over time on Hilbre Island. Individual models were run for each climate variable: rainfall, temperature and snowfall. Coefficients in bold had estimate directions supported by > 95% of the posterior distribution. Response variables were not standardised and are reported in their original units (mm of rainfall, °C of temperature, mm/cm of snowfall) to maintain interpretability in terms of actual climate measurements. Therefore, the intercepts reflect the mean value of the climate variables in their raw units.
**Figure S1:** The East Atlantic Flyway. Image by CWSS, taken and adapted from van Roomen et al. (2022): “Birds from the Arctic (in orange) breeding between Siberia and Northeast Canada use sites (blue dots) along the Eastern shore of the Atlantic Ocean and connect these during migration and wintering. At these sites they mix up with breeding populations both from Western Europe and Western Africa which also migrate and winter at these sites. Hilbre Island (green dot) is among the UK‐based breeding sites identified (blue dots).
**Figure S2:** Posterior predictive checks for the three climate models. Dark blue lines represent the observed data; the light blue lines represent 100 draws from the posterior.
**Figure S3:** Posterior predictive checks for the phenology and abundance models. Dark blue lines represent the observed data; the light blue lines represent 100 draws from the posterior.
**Figure S4:** Effects of year on climate variables at Hilbre Island. Coloured lines show model‐predicted values for rainfall (blue), temperature (red) and snowfall (purple) over time, with semi‐transparent ribbons representing 95% confidence intervals. Grey points show observed yearly values in their original units (rainfall in mm, temperature in °C, snowfall in mm). Response variables were not standardised; intercepts reflect mean values over the study period. Correlation coefficients between each variable and year are displayed in each panel. Y‐axis scales are independent to capture the observed ranges without exaggerating effect sizes, and visit years were centred in the models to make small slopes visually apparent.
**Figure S5:** Correlations between yearly averages on Hilbre and UK‐wide yearly averages for all climatic variables included in our study (*n* = 46 years).

## Data Availability

The data used in this study can be accessed here: https://doi.org/10.5061/dryad.h44j0zq04.
