# Supplementary material for: Fifty Years of Bird Ringing Reveal Opposing Seasonal Responses of Migration Timing to Temperature and Rainfall on Hilbre Island
Source: Ecol Evol. 2026 Apr 5;16(4):e73377. doi: 10.1002/ece3.73377 (PMC13051806; doi:10.1002/ece3.73377)
Supplement: Supplementary file 1 — Table S1: Individual species included in data analysis based on selection criterion described in the methods. Status is based on historical records of bird activity on Hilbre Island. Species in shaded cells appear in all models; the remaining species were included in our analyses of abundance but arrival dates or residence in spring and autumn. Table S2: Model results exploring climate change over time on Hilbre Island. Individual models were run for each climate variable: rainfall, temperature and snowfall. Coefficients in bold had estimate directions supported by > 95% of the posterior distribution. Response variables were not standardised and are reported in their original units (mm of rainfall, °C of temperature, mm/cm of snowfall) to maintain interpretability in terms of actual climate measurements. Therefore, the intercepts reflect the mean value of the climate variables in their raw units. Figure S1: The East Atlantic Flyway. Image by CWSS, taken and adapted from van Roomen et al. (2022): “Birds from the Arctic (in orange) breeding between Siberia and Northeast Canada use sites (blue dots) along the Eastern shore of the Atlantic Ocean and connect these during migration and wintering. At these sites they mix up with breeding populations both from Western Europe and Western Africa which also migrate and winter at these sites. Hilbre Island (green dot) is among the UK‐based breeding sites identified (blue dots). Figure S2: Posterior predictive checks for the three climate models. Dark blue lines represent the observed data; the light blue lines represent 100 draws from the posterior. Figure S3: Posterior predictive checks for the phenology and abundance models. Dark blue lines represent the observed data; the light blue lines represent 100 draws from the posterior. Figure S4: Effects of year on climate variables at Hilbre Island. Coloured lines show model‐predicted values for rainfall (blue), temperature (red) and snowfall (purple) over time, with semi‐transpare [file ECE3-16-e73377-s001.docx]

**Supplementary material**

**Table S1.** Individual species included in data analysis based on selection criterion described in the methods. Status is based on historical records of bird activity on Hilbre Island. Species in shaded cells appear in all models; the remaining species were included in our analyses of abundance but arrival dates or residence in spring and autumn.

| **BTO CODE** | **SPECIES** | **ORDER** | **STATUS** | **SEASON** |
| --- | --- | --- | --- | --- |
| BLABI | Blackbird | Passeriformes | Resident and Migratory | Year-round |
| BLACA | Blackcap | Passeriformes | Migratory | Spring |
| BLUTH | Bluethroat | Passeriformes | Migratory | Spring |
| BLUTI | Blue tit | Passeriformes | Migratory | Autumn |
| CHAFF | Chaffinch | Passeriformes | Migratory | Autumn |
| CHIFF | Chiffchaff | Passeriformes | Migratory | Spring |
| DUNLI | Dunlin | Charadriiformes | Migratory | Autumn |
| DUNNO | Dunnock | Passeriformes | Resident and Migratory | Year-round |
| FIELD | Fieldfare | Passeriformes | Migratory | Autumn |
| FIREC | Firecrest | Passeriformes | Migratory | Autumn |
| GARWA | Garden warbler | Passeriformes | Migratory | Spring |
| GOLDC | Goldcrest | Passeriformes | Migratory | Autumn |
| GOLDF | Goldfinch | Passeriformes | Migratory | Autumn |
| GRAWA | Grasshopper warbler | Passeriformes | Migratory | Spring |
| GREFI | Greenfinch | Passeriformes | Migratory | Autumn |
| HOUSP | House sparrow | Passeriformes | Resident and Migratory | Year-round |
| KNOT | Knot | Charadriiformes | Migratory | Autumn |
| LESRE | Lesser redpoll | Passeriformes | Migratory | Autumn |
| LINNE | Linnet | Passeriformes | Resident and Migratory | Year-round |
| LOTTI | Long-tailed tit | Passeriformes | Migratory | Autumn |
| MARWA | Marsh warbler | Passeriformes | Migratory | Spring |
| MEAPI | Meadow pipit | Passeriformes | Migratory | Autumn |
| MELWA | Melodious warbler | Charadriiformes | Migratory | Spring |
| OYSTE | Oystercatcher | Charadriiformes | Migratory | Autumn |
| PIEWA | Pied wagtail | Passeriformes | Migratory | Spring |
| PURSA | Purple sandpiper | Charadriiformes | Migratory | Autumn |
| REDSH | Redshank | Charadriiformes | Migratory | Autumn |
| REDST | Common redstart | Passeriformes | Migratory | Spring |
| REDWI | Redwing | Passeriformes | Migratory | Autumn |
| REEBU | Reed bunting | Passeriformes | Migratory | Autumn |
| RINPL | Ringed plover | Charadriiformes | Migratory | Autumn |
| ROBIN | European robin | Passeriformes | Resident and Migratory | Year-round |
| ROCPI | Rock pipit | Passeriformes | Resident and Migratory | Year-round |
| SANDE | Sanderling | Charadriiformes | Migratory | Autumn |
| SEDWA | Sedge warbler | Passeriformes | Migratory | Spring |
| SISKI | Siskin | Passeriformes | Migratory | Autumn |
| SKYLA | Skylark | Passeriformes | Migratory | Autumn |
| SONTH | Song thrush | Passeriformes | Migratory | Autumn |
| SPARR | Sparrowhawk | Accipitriformes | Migratory | Autumn |
| SPOFL | Spotted flycatcher | Passeriformes | Migratory | Spring |
| STARL | Starling | Passeriformes | Migratory | Autumn |
| SWALL | Swallow | Passeriformes | Migratory | Spring |
| TRESP | Tree sparrow | Passeriformes | Migratory | Autumn |
| TURNS | Turnstone | Charadriiformes | Migratory | Autumn |
| WHEAT | Wheatear | Passeriformes | Migratory | Spring |
| WHINC | Whinchat | Passeriformes | Migratory | Spring |
| WHITE | Whitethroat | Passeriformes | Migratory | Spring |
| WILWA | Willow warbler | Passeriformes | Migratory | Spring |
| WREN | Wren | Passeriformes | Resident and Migratory | Year-round |

**Table S2.** Model results exploring climate change over time on Hilbre Island. Individual models were run for each climate variable: rainfall, temperature and snowfall. Coefficients in bold had estimate directions supported by >95% of the posterior distribution. Response variables were not standardised and are reported in their original units (mm of rainfall, °C of temperature, mm/cm of snowfall) to maintain interpretability in terms of actual climate measurements. Therefore, the intercepts reflect the mean value of the climate variables in their raw units.

| **VARIABLE** | **CO-EFFICIENT** | **ESTIMATE** | **ESTIMATE ERROR** | **L-95% CI** | **U-95% CI** |
| --- | --- | --- | --- | --- | --- |
| Rain | Intercept | -199.522 | 192.094 | -575.736 | 175.273 |
| Rain | Visit year | 0.132 | 0.096 | -0.056 | 0.321 |
| **Temp** | **Intercept** | **-46.754** | **10.777** | **-67.748** | **-24.865** |
| **Temp** | **Visit year** | **0.029** | **0.005** | **0.018** | **0.039** |
| Snow | Intercept | 78.079 | 126.032 | -172.580 | 326.668 |
| Snow | Visit year | -0.037 | 0.063 | -0.162 | 0.089 |

**
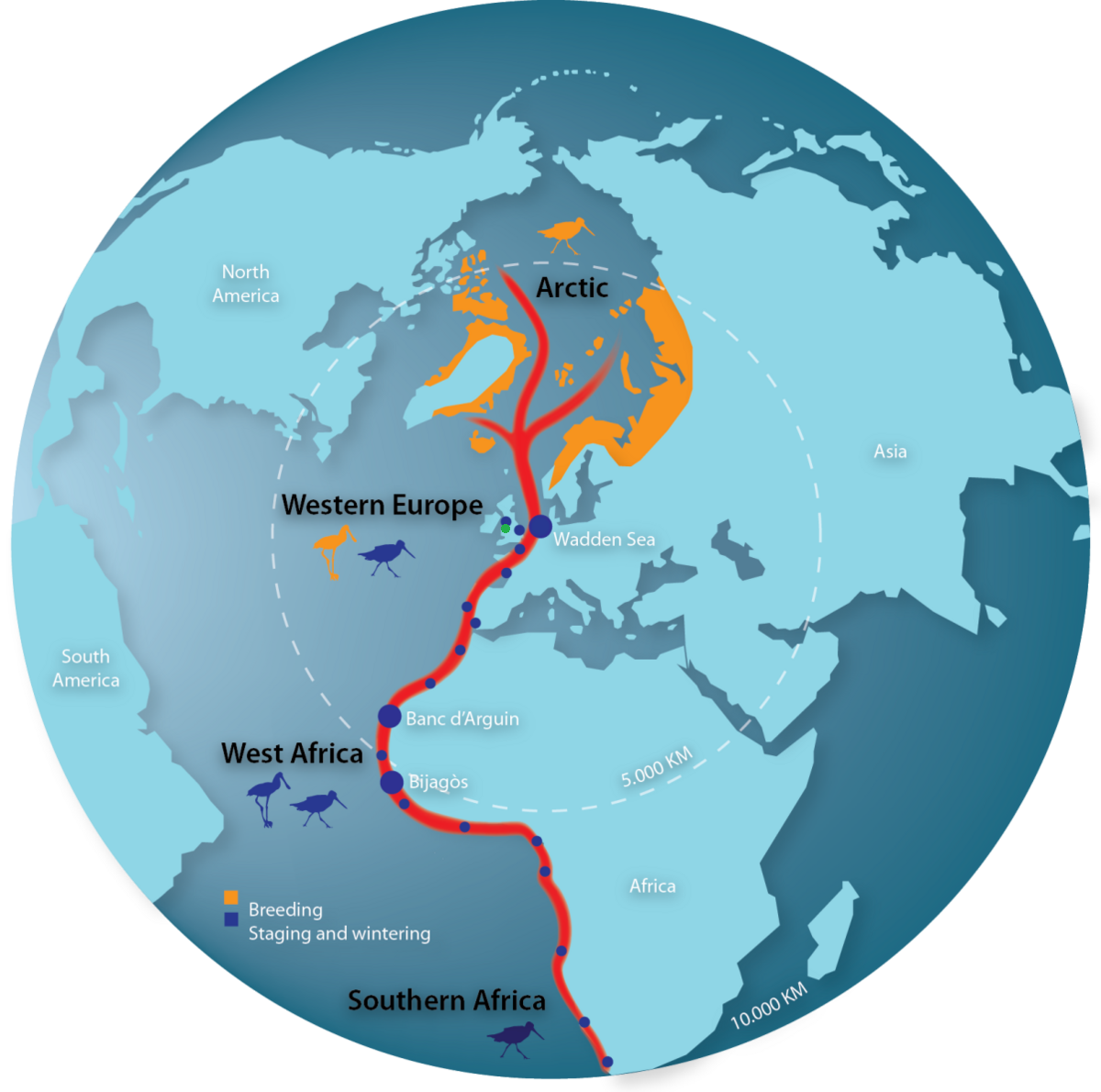
**

**Figure S1.**  The East Atlantic Flyway. Image by CWSS, taken and adapted from van Roomen et al. (2022): “Birds from the Arctic (in orange) breeding between Siberia and Northeast Canada use sites (blue dots) along the Eastern shore of the Atlantic Ocean and connect these during migration and wintering. At these sites they mix up with breeding populations both from Western Europe and Western Africa which also migrate and winter at these sites. Hilbre Island (green dot) is among the UK-based breeding sites identified (blue dots).


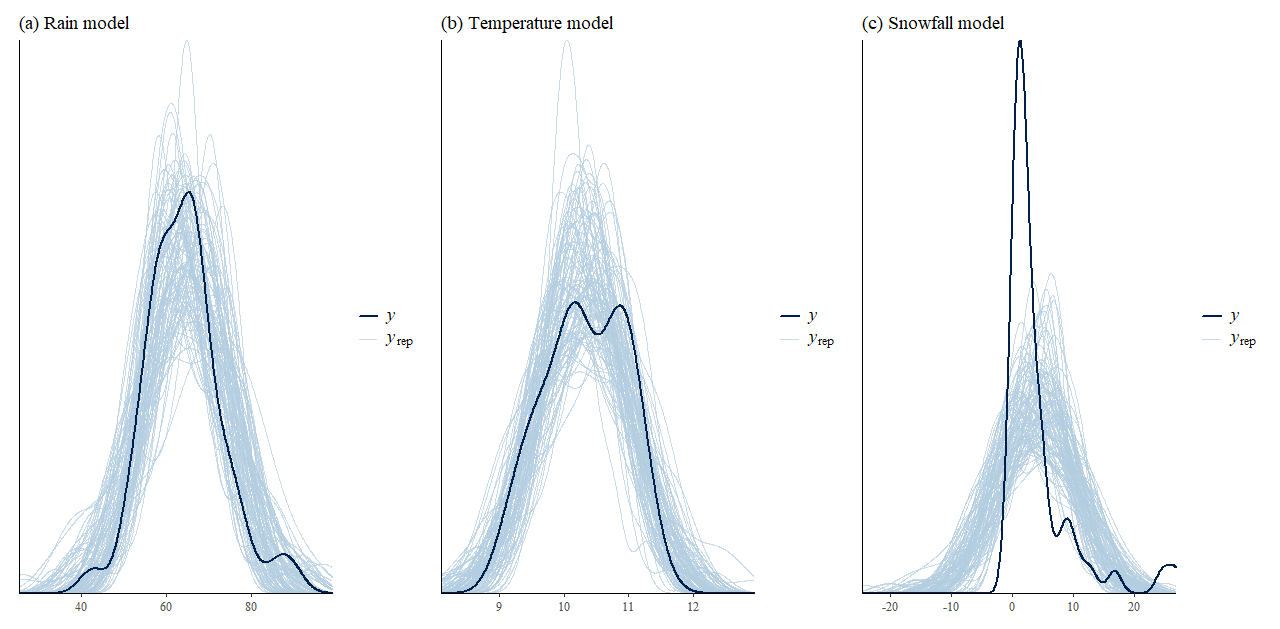


**Figure S2.** Posterior predictive checks for the three climate models. Dark blue lines represent the observed data; the light blue lines represent 100 draws from the posterior.


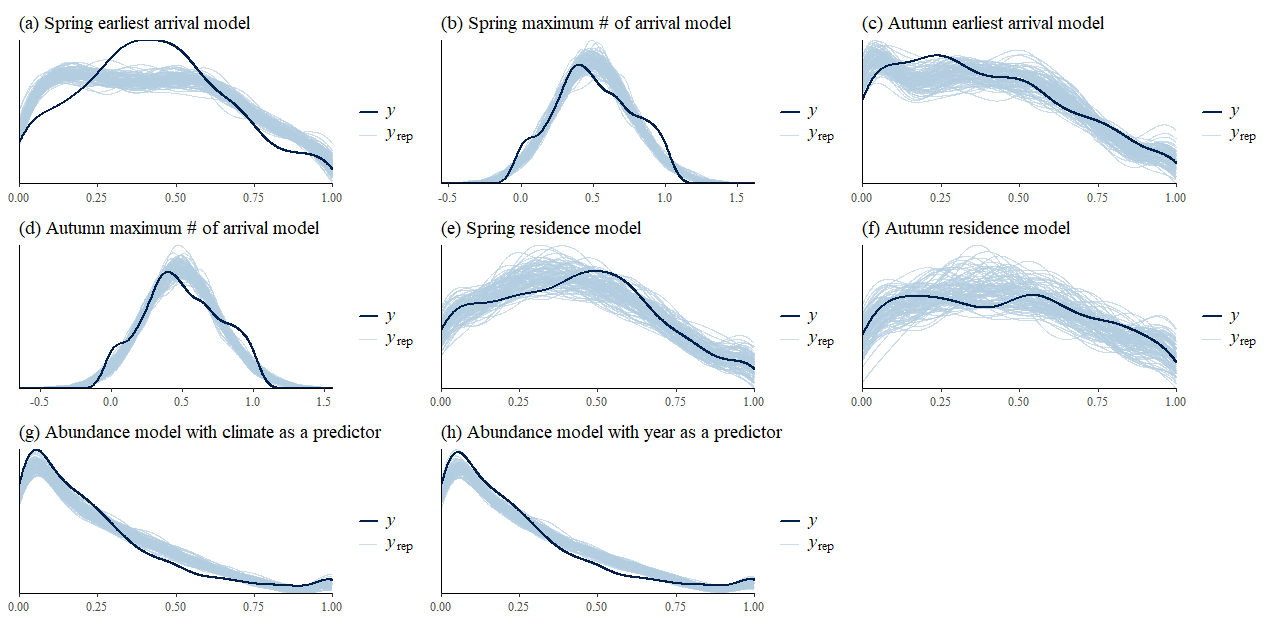


**Figure S3.** Posterior predictive checks for the phenology and abundance models. Dark blue lines represent the observed data; the light blue lines represent 100 draws from the posterior.


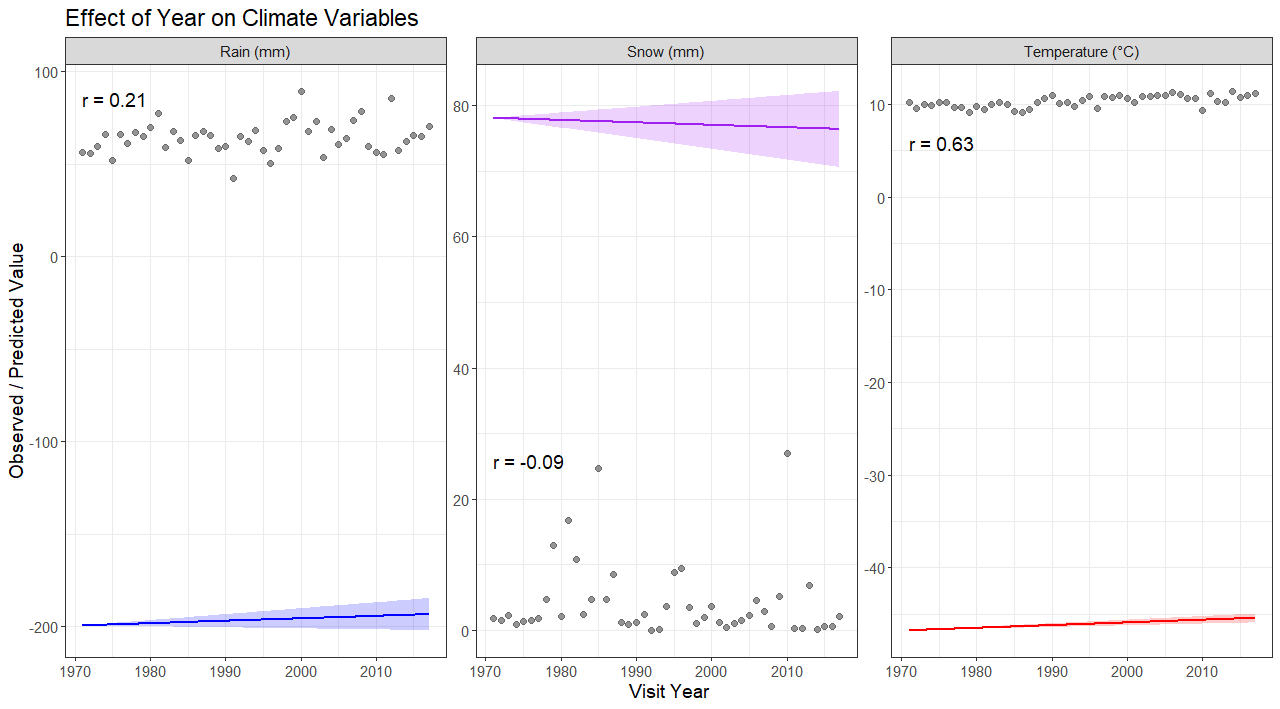


**Figure S4.** Effects of year on climate variables at Hilbre Island**.** Coloured lines show model-predicted values for rainfall (blue), temperature (red), and snowfall (purple) over time, with semi-transparent ribbons representing 95% confidence intervals. Grey points show observed yearly values in their original units (rainfall in mm, temperature in °C, snowfall in mm). Response variables were not standardised; intercepts reflect mean values over the study period. Correlation coefficients between each variable and year are displayed in each panel. Y-axis scales are independent to capture the observed ranges without exaggerating effect sizes, and visit years were centred in the models to make small slopes visually apparent.


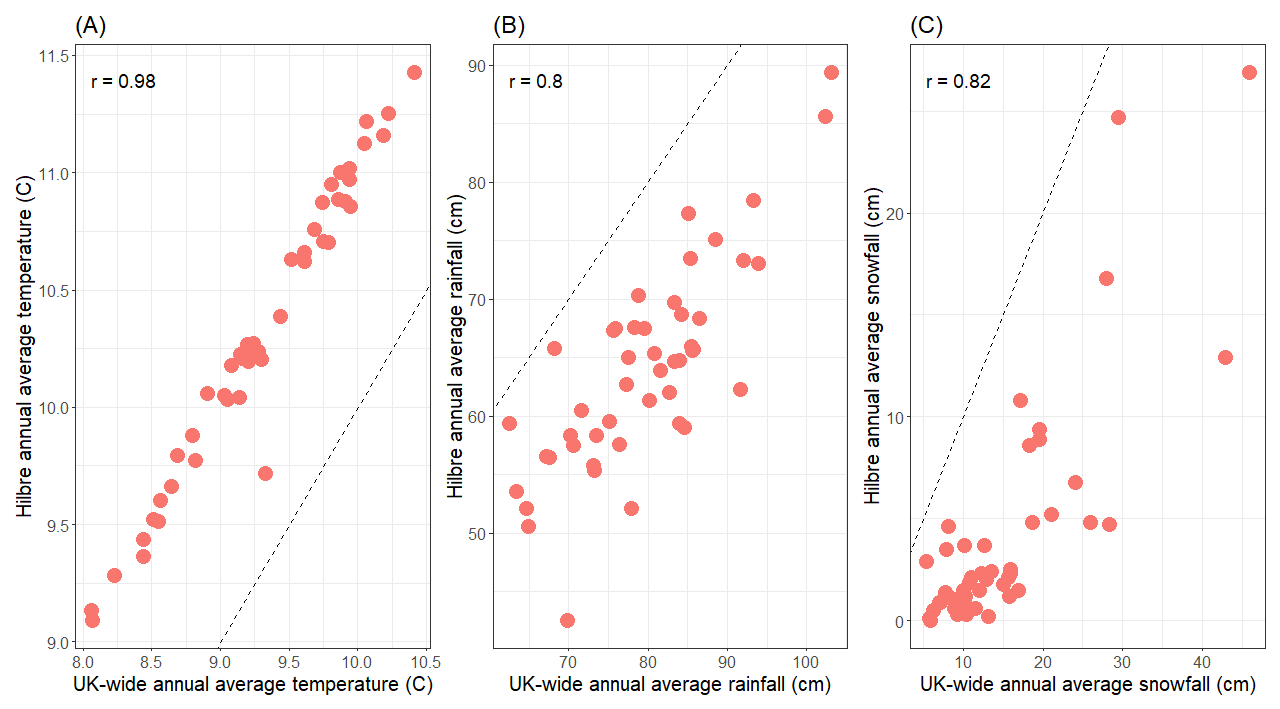


**Figure S5.** Correlations between yearly averages on Hilbre and UK-wide yearly averages for all climatic variables included in our study (n=46 years).
